# Editorial: Advancements in computational studies of drug toxicity

**DOI:** 10.3389/fphar.2023.1230409

**Published:** 2023-06-06

**Authors:** Noah R. Flynn, Grover P. Miller, S. Joshua Swamidass

**Affiliations:** ^1^ Alexa AI, Amazon, Cambridge, MA, United States; ^2^ Department of Biochemistry and Molecular Biology, College of Medicine, University of Arkansas for Medical Sciences, Little Rock, AR, United States; ^3^ Department of Pathology and Immunology, Washington University School of Medicine, St. Louis, MO, United States

**Keywords:** machine learning, deep learning, drug toxicity, adverse drug reactions, QSAR, mutagenicity, computational chemistry, cardiotoxicity

Computational approaches to studying drug toxicity have been and continue to be an important tool in drug discovery. *In silico* methods are especially appealing for their ease of use and reduction of resources and expenses. Advances in machine learning and deep learning have made substantial advances in recent years, offering new applications in the study of drug toxicity. Drug toxicity concerns (e.g., mutagenicity, endocrine toxicity, or cardiotoxicity) identified during screening and optimization can prevent otherwise strong therapeutic candidates from proceeding. Inability to satisfy drug safety criteria is a primary driver of drug withdrawal and termination during clinical development. Our goal is to review key advances in computational studies of drug toxicity, especially those that may be pragmatically useful in drug discovery.

In this Research Topic, five unique advancements in computational studies of drug toxicity have been published. We present each accepted publications in chronological order:

1) *A comparison of nine machine learning mutagenicity models and their application for predicting pyrrolizidine alkaloids* (Helma et al.). In this study, the authors take an important step in understanding how *in silico* approaches could help overcome practical limitations of *in vitro* testing the abundance of pyrrolizidine alkaloid (PA) compounds. They compiled a new, publicly available mutagenicity training dataset focused on *Salmonella typhimurium*. With that dataset, they compared the applicability of trained models to identify structural features driving mutagenicity among PAs across model type (e.g., neural networks, random forest, support vector machine, logistic regression, and k-nearest neighbor) and descriptor type (MolPrint2D or Chemistry Development Kit). The approach demonstrated a common clustering of PA subgroups that may provide a way to tier rank mutagenic risk.
2) *Quantitative structure-activity relationship modeling of the amplex ultrared assay to predict thyroperoxidase inhibitory activity* (Gadaleta et al.). Here, the authors present a valuable advancement in modeling thyroid pathologies (e.g., hyperthyroidism and hypothyroidism) induced by xenobiotics and other hazardous contaminants. The researchers used data on thyroid peroxidase inhibition to develop a QSAR model involving consensus predictions from balanced random forest and k-nearest neighbors algorithms. Their models provided a useful way to rank toxic risks posed by the environmental chemicals toward disruptions in the thyroid system as a basis for mitigating those risks.
3) *Path4Drug: data science workflow for identification of tissue-specific biological pathways modulated by toxic drugs* (Füzi et al.). The authors have designed and released an open-source computational pipeline, Path4Drug, that uses drug-protein interactions to identify biological pathways perturbed by a drug. They validated their workflow in the context of withdrawn drugs and cardio- and hepatotoxic drugs with black box warnings, connecting known toxic events to biological pathways that those drugs may alter. Moreover, their approach identified other, unstudied pathways as possible targets for further study. Path4Drug is openly available, modifiable, and adaptable to applications to other systems biology contexts.
4) *Ligand-based prediction of herg-mediated cardiotoxicity based on the integration of different machine learning techniques* (Delre et al.). In this work, the authors succeed in ensuring availability of a novel, robust ligand-based workflow for predicting hERG-related cardiotoxicity. Furthermore, the authors covered the performance of 30 ligand-based classifiers of human-ether-a-go-go (hERG) related cardiotoxicity. Notably, they determined optimal choices for data curation, feature selection, sampling, and model type to assemble a freely available computational workflow that outperformed several commonly employed and freely available techniques.
5) *Ensemble of structure and ligand-based classification models for herg liability profiling* (Vittorio et al.). In the final work of this Research Topic, Vittorio et al. develop several random forest models for assessing hERG-related cardiotoxicity, which is of significant interest for its role as an anti-target in drug candidate screening. Their experimental approach involved a helpful comparison of performances of two common but competing ligand- and protein-based model designs for predicting those liabilities. With the same dataset, ligand-based approach was the best overall but the combination of the models seem more useful for new, untested molecules.

These five publications showcase the usefulness of machine learning in advancing computational studies of drug toxicity. Many research groups involved in drug discovery can benefit from inclusion of the machine learning methods discussed throughout this Research Topic. In particular, the described approaches are extensible to common toxicity concerns including bioactivity, drug-drug interactions, and drug-induced liver injury. We hope that this article Research Topic inspires readers to embark on new possibilities offered by growing applications of artificial intelligence to drug toxicity and their relevance during lead-finding, lead-optimization, screening, and modeling of metabolism and pharmacology.

